# Effect of Supplementing Dairy Goat Diets With Rapeseed Oil or Sunflower Oil on Performance, Milk Composition, Milk Fatty Acid Profile, and *in vitro* Fermentation Kinetics

**DOI:** 10.3389/fvets.2022.899314

**Published:** 2022-06-16

**Authors:** Einar Vargas-Bello-Pérez, Nanna Camilla Pedersen, Jaloliddin Khushvakov, Yongxin Ye, Rajan Dhakal, Hanne H. Hansen, Lilia Ahrné, Bekzod Khakimov

**Affiliations:** ^1^Department of Veterinary and Animal Sciences, Faculty of Health and Medical Sciences, University of Copenhagen, Frederiksberg, Denmark; ^2^Department of Food Science, Faculty of Science, University of Copenhagen, Frederiksberg, Denmark; ^3^Institute of Chemistry and Biotechnology, Zurich University of Applied Sciences, Winterthur, Switzerland

**Keywords:** goats, milk, oilseeds, rumenic acid, unsaturated fatty acids, vaccenic acid

## Abstract

The aim of this study was to determine the effect of supplementing dairy goat diets with rapeseed oil and sunflower oil on performance, milk composition, milk fatty acid profile, and *in vitro* fermentation kinetics. Nine Danish Landrace goats with 42 ± 5 days in milk were allocated to three treatment groups for 42 days. Animals received a basal diet, formulated with 85:15 forage:concentrate ratio, and the basal diet was supplemented with either rapeseed oil or sunflower oil at 4% of dry matter. Goat milk was sampled on days 14, 21, and 42. Milk composition was similar between treatments. From day 14 to day 42, milk yield increased (1.03 vs. 1.34 kg/d), while milk fat (2.72 vs. 1.82 g/d) and total solids (11.2 vs. 9.14 %) were reduced. Compared to control and rapeseed oil, sunflower decreased (*P* < 0.05) C4:0 (1.56, and 1.67 vs. 1.36 g/100 g) and both oils decreased (*P* < 0.05) C18:3n3 (0.60 vs. 0.20 and 0.10 g/100g). Rapeseed oil increased (*P* < 0.05) C18:2 *cis*9, *trans*11 compared to control and sunflower oil (0.37 vs. 0.13 and 0.19 g/100 g). Untargeted milk foodomics revealed slightly elevated (*P* < 0.05) gluconic acid and decreased hippuric acid (*P* < 0.05) in the milk of oil-fed goats compared to control. *In vitro* dry matter degradation (63.2 ± 0.02 %) was not affected by dietary treatments, while individual volatile fatty acid proportions, total volatile fatty acids (35.7 ± 2.44 mmol/l), CO_2_ (18.6 ± 1.15 mol), and CH_4_ (11.6 ± 1.16 mol) were not affected by dietary treatments. Sunflower oil and rapeseed oil decreased (*P* < 0.05) total gas production at 24 and 48 h compared with control. Overall, the use of sunflower oil or rapeseed oil at 4% DM inclusion did not compromise animal performance and milk composition.

## Introduction

Goat milk fat content and its chemical composition has been extensively modulated by nutritional and genetic factors (1, 2). The most practical way to modulate milk fatty acids (FA) toward a healthier profile for human consumption is by supplementing animals with vegetable lipid sources (3, 4). However, responses will depend on individual FA chain length, degree of saturation, and stereochemistry of double bonds as previously shown at *in vitro* level in goat mammary gland cells (5). When ruminants are fed with unsaturated FA sources, milk FA profiles are changed because of rumen biohydrogenation, whereby dietary unsaturated FA undergoes hydrogenation and isomerization (6).

Dietary lipids induce changes during rumen fermentation (7) and those could lead to decrease contents of volatile fatty acids (VFA), CO_2_, and CH_4_ (8). Dietary fat reduces CH_4_ production in the rumen by reducing hydrogen accumulation through FA biohydrogenation, reducing the intake of fermentable organic matter, reducing fiber digestion, and inhibiting the activity of ruminal methanogens (9). This knowledge has been used to design nutritional strategies that include fat and reduce CH_4_ production by dairy animals.

Consumers are aware that saturated fats from dairy products could have negative effects on their health (10) and this has led to the design of diets where unsaturated fats are added to improve milk fat quality. For example, in goats, dietary supplementation of rapeseed oil (11) and sunflower oil decrease milk contents of saturated fatty acids (12, 13). Currently, it has been reported that milk foodome could be changed by animal species or feeding regime, which has been explored previously in goats and cows (14, 15).

The possible effects of supplementing rapeseed or sunflower oils in the goat milk foodome have been investigated (16), reporting that feeding goats with sunflower oil can increase relative concentrations of milk amino acids and organic acids while rapeseed oil increased aliphatic alcohols, including ethanol and organic acids. However, differently from the previous study, this study shows the effect of supplementing dairy goat diets with rapeseed oil or sunflower oil on milk yield, fatty acid profile, and *in vitro* fermentation kinetics. For this purpose, retail sunflower oil was chosen as a source of monounsaturated FA (61 g /100 g of oleic acid) and retail rapeseed oil as a source of polyunsaturated FA (60 g /100 g of linoleic acid). Therefore, it is expected that these oilseed oils will exert differential effects on goat lipid metabolism as they have different numbers and locations of double bonds in their FA structure.

## Materials and Methods

The present study was conducted in compliance with the Danish Ministry of Justice Law No. 474 (15 May 2014) concerning animal experimentation and the care of experimental animals. The Animal Ethics Institutional Review Board from the University of Copenhagen approved this study (ID 2021-08-PNH-008A).

### Animals and Diets

The study was performed at a goat farm located in Tureby, Denmark (55°19'38.9”N, 12°06'36.1”E). Nine Danish Landrace goats with twin kids, with 42 ± 5 days in milk at the beginning of the study were allocated to three treatment groups. At the onset of the study average body condition score for the 3 groups were 3.2 ± 0.2, 3.0 ± 0.1, and 2.8 ± 0.2 (scored on a five-point scale) while body weights were 45.4 ± 4.9, 47 ± 11, and 40.1 ± 6.3. Goats were group-housed with their kids in stalls (12 × 38 m) with continuous access to water. Body condition score (BCS; scored on a five-point scale where 1 = emaciated to 5 = overly fat) and body weight were recorded on days 14, 21, and 42.

For 42 days, all animals received a common forage portion (Table 1). Forage was offered daily at 0700 h and the concentrate (300 g/d/animal) was supplied during milking at 13:00 h. Dietary treatments contained 85:15 forage to concentrate ratio. Diets were formulated to meet the nutrient requirements of a mature dairy doe with twin kids and a body weight between 40 and 50 kg according to the NRC (17). Diets were planned to supply a dry matter intake of 2.39 kg/d, 3.38 Mcal/d of metabolizable energy, and degradable intake protein of 84 g/d. The control concentrate consisted of (% of dry matter) 93% of a grain mix (rolled barley, rolled oats, rolled wheat, and peas), 6% of molasses and 1% of a premix of vitamins and minerals. Oils-supplemented concentrates contained 89% of the grain mix, 6% of molasses, 1% of a premix of vitamins and minerals, and 4 % of either sunflower oil or rapeseed oil. Oils were not rumen protected and were mixed manually and homogenously into the daily concentrate of each goat.

**Table 1:** Diet and treatments composition.

	**Diets**		
	**Control**	**Rapeseed oil**	**Sunflower oil**
**Forage (%)**			
Alfalfa + grass hay	42	42	42
Clover haylage	23	23	23
Alfalfa + clover hay	21	21	21
Straw	14	14	14
**Concentrate (%)**			
Rolled barley	37	37	37
Rolled oats	28	28	28
Rolled wheat	19	19	19
Rolled peas	9	9	9
Molasses	6	6	6
Vitamins and minerals premix	1	1	1
Rapeseed oil	0	4	0
Sunflower oil	0	0	4
**Chemical composition, % DM**			
Dry matter of diet	82.5	83.2	82.8
Ether extract	2.39	6.92	6.93
Crude protein	1.40	1.38	1.35
Ash	3.18	3.18	3.40
Neutral detergent fiber	55.3	55.3	55.3
Acid detergent fiber	32.3	32.3	32.3
Lignin	4.8	4.9	4.8
**Fatty acids, g/100 g**			
C16:0	0.6	5.4	4.1
C18:0	1.0	3.5	0.7
C18:1	18.0	24.5	54.0
C18:2	45.6	56.1	24.7
C18:3	32.6	9.5	15.8
>C20	2.2	0.9	0.8

Diets were analyzed following standard procedures from AOAC (18) to determine dry matter (934.01) and Kjeldahl N (984.13). Ash and ether extract were analyzed according to AOAC [(19); methods 942.05 and 920.39, respectively]. Neutral detergent fiber, acid detergent fiber, and acid detergent lignin were determined following Van Soest et al. (20). The chemical composition of dietary treatments is shown in Table 1.

### Milk Production and Composition

Milk yield was recorded at 07:00 h on days 14, 21, and 42 prior to a 12 h separation of does and their kids. Milk samples (50 ml) were pooled for each collection period and treatment and were analyzed for fat, protein, lactose, casein, total solids, citric acid, solids-not-fat, urea, free fatty acid, acidity, and density. These analyses were done in duplicate by Fourier Transform Infrared Spectroscopy using a MilkoScan™ FT2 (Foss Analytical A/S, Hillerød, Denmark). For statistical analysis, means across replicates were calculated and used. Individual milk samples (200 ml) were taken at 10:00 h on days 14, 21, and 42. All raw milk samples were stored at −4°C for further FA analysis while milk samples pooled by treatment were analyzed for foodome using gas chromatography–mass spectrometry (GC–MS) and proton (^1^H) nuclear magnetic resonance (NMR) spectroscopy.

### Milk Fatty Acid Analysis

Lipid extraction and methylation of plasma samples were done as reported previously by Vargas-Bello-Pérez (21). A gas chromatograph (GC-2030) system (Shimadzu Scientific Instruments AOC-20i Plus, Columbia, MD, USA) equipped with a 100-m column (Rt-2560 column 100 m × 0.32 mm × 0.20 μm column; Restek, Bellefonte, PA, USA) was used with the following conditions after injection, the oven temperature was set at 110°C for 4 min and then raised to 170°C at a rate of 5°C/min for 10 min, to 225°C at 3°C/min for 10 min, and finally increased to 240°C at 3°C/min. The temperature of the ionization flame was 260°C, injection volume was 2 μl, hydrogen flow was 32 ml/min, air flow was 200 ml/min, and nitrogen flow was 24 ml/min. The total running time was 59.33 min. Fatty acid peaks were identified using a fatty acid methyl ester (FAME) standard (Supelco 37 Component FAME mix, Bellefonte, PA, USA). Reference standards for C18:1, *trans*11 and C18:1 *cis*9, *trans*11 (Nu-Chek Prep Inc., Elysian, MN, USA) methyl esters were used.

### Milk Foodome

Milk foodome was measured as previously described using GC–MS and ^1^HNMR spectroscopy (16). Briefly, frozen milk samples were thawed at room temperature and vigorously vortexed until homogenization. After sonication, 200 μl milk was mixed with 300 μl of 80% methanol (containing 10 ppm internal standard, sorbitol) and 100 μl dichloromethane and vigorously vortexed followed by centrifugation at 13,572 g for 10 min at 4°C. Then 100 μl upper aqueous layer was transferred into a 200 μl glass insert and dried overnight using ScanVac (Labogene, Lynge, Denmark) at 40°C and 1,000 rpm. Immediately after drying, glass inserts were sealed with airtight magnetic lids into GC–MS vials, stored at 4°C,and analyzed by GC–MS within 24 h. For ^1^H NMR analysis, frozen milk samples were thawed at room temperature, vigorously vortexed until homogenization and 1.8 ml of milk samples were centrifuged at 13,572 g for 30 min at room temperature. Then, 600 μl of an aliquot from the clear solution was mixed with 135 μl of phosphate buffer in deuterated water and transferred into NMR SampleJet tubes of L = 103.5 mm and O.D. = 5.0 mm, kept at 5°C and analyzed within 24 h. GC–MS and ^1^H NMR datasets were subsequently processed using PARADASe (22) or SigMa (23) software, respectively, to convert the raw milk foodomics data into an informative metabolite table.

### *In vitro* Fermentation Kinetics

Two 48-h *in vitro* fermentations were undertaken with four technical replicates of each treatment. Equal amounts of rumen fluid were taken from two cannulated Jersey heifers, owned by the Large Animal Hospital at the University of Copenhagen, and licensed according to Danish law (authorization nr.2012-15-2934- 00648). The cannulated heifers were fed a maintenance level diet consisting of *ad libitum* haylage (85% DM; 7.5 MJ/kg DM; 11% CP), for 6 weeks before sampling.

A 500 mg sample from each of the freeze-dried treatments to be tested was weighed into each of four 100 ml Duran® bottles. Each bottle was fitted with an automatic wireless *in vitro* gas production module (Ankom Technology, Macedon, NY, U.S.A.) with a pressure sensor (pressure range: from −69 to +3,447 kPa; resolution: 0.27 kPa; accuracy: ± 0.1% of measured value). Each module sends measurements via a receiving base station to an attached computer. The accumulated gas was automatically released (250 ms vent opening) when the pressure inside the units reached 0.75 PSI above the ambient pressure. The absolute pressure was recorded every 10 min to calculate cumulative pressure and later converted to milliliter using the ideal gas law. A buffer solution was prepared as described by Menke et al. (24). The buffer media was flushed with CO_2_ for 2 h to ensure anaerobic conditions and the temperature of the media was maintained at 39°C before the addition of rumen fluid. A reduction agent of sodium sulfide and sodium hydroxide was added 10 min before the addition of the rumen fluid.

The rumen content, including liquid and particulates, was collected before morning feeding, and transported to the laboratory in preheated thermos bottles. Rumen fluid was filtered through two layers of cheesecloth to eliminate large feed particles, and the particulate material was squeezed to collect microbes attached to feed particles. The rumen fluid was added to the buffer in a 1:2 ratio. A total of 90 ml of this inoculum was added to each bottle after which the headspace of each bottle was flushed with nitrogen, closed with the module head, and incubated in a Thermoshaker at 39.5°C for 48 h. The initial pH of the inoculum was 6.97. After 48 h of incubation, the modules were put into an ice bath to stop fermentation, and pH was measured from each bottle. The residual material in each bottle was filtered into a weighed ANKOM F57 filter bag (Ankom Technology, Macedon, NY, USA). Thereafter, the bag and residue were dried, weighed, and burned at 550°C for 12 h. The weight of the residue was used to determine organic matter (OM) degradation. Two bottles with no feed sample but rumen inoculum followed the entire procedure to correct for total gas, residual dry matter, and ash from the rumen fluid. The pressure of the accumulated gas was converted into volume (ml) per gram OM sample at standard pressure and temperature (STP).

Rumen fluid samples were preserved for VFA determination by adding 25% metaphosphoric acid in a 5:1 ratio. Samples were frozen (−20°C) for later analysis. The VFA was determined by gas chromatography (Nexis GC-2030, Shimadzu Scientific Instruments Inc., Kyoto, Japan) equipped with a 30-m wall-coated open-tubular fused-silica capillary column (Stabilwax-DA; 30 m × 0.32 mm i.d., 0.25 μm film thickness; Shimadzu, USA). The running time per sample was 8.71 min. The oven temperature was programmed for 145°C for 3 min and then increased from 145°C to 245°C at 16.6°C/min. The injector and flame ionization detector were maintained at 250°C. Gas flows were 24, 32, and 200 ml/min for N_2_, H_2_, and air, respectively.

The following calculations were performed to determine fermentative CO_2_ and CH_4_ as described by Wolin (25) and Makkar (26):


$$\begin{array}{l} \text{Fermentative C}{\text{O}}_{2}=\text{A}/2+\text{P}/41+\;1.5\text{B} \end{array}$$

where A, P, and B are moles of acetic acid, propionic acid, and butyric acid, respectively.


$$\begin{array}{l} \text{Fermentative C}{\text{H}}_{4}=\;\left(\text{A}+2\text{B}\right)-\text{C}{\text{O}}_{2} \end{array}$$

where A and B are moles of acetic acid and butyric acid, respectively and CO_2_ is moles of CO_2_ calculated from the previous equation.

### Statistical Analysis

Data on milk yield, milk composition, and milk FA profile were analyzed using the MIXED procedure in SAS (SAS Institute Inc., Cary, NC). A model including diet, time, and diet × time as fixed effects and goat within treatment as a random effect was used. Least squares means (LSM) were separated using the PDIFF (Piecewise Differentiable) statement in SAS.

Fermentation data were analyzed using the statistical program R (27, 28). A linear mixed model was used for *in vitro* data. The normality of the model was accessed using quantile–quantile plots of the model. ANOVA was used to access the significance of the predictor of the model. Significant differences between each of the individual treatments were determined by using Tukey's Honest Significance difference test and significance was considered at *p* < 0.05. All the results are expressed as least-square means and standard error of the mean. A correlation analysis was performed to see the correlation between different fatty acids. The following model was used to run the analysis for *in vitro* fermentation parameters.


$$\begin{array}{l} \text{Y }=\text{ Treatment }+\text{Random}\left(\text{Run}\right)\;+\text{Error} \end{array}$$

A one-way ANOVA with Benjamini–Hochberg's multiple test correction approaches (29) using a false discovery rate (FDR) of 5% was applied to investigate the possible effect of dietary treatments on milk metabolites as measured by GC–MS and ^1^H NMR. Foodomics data analysis was performed in MATLAB (MathWorks Inc., Massachusetts, USA) using customized scripts written by the authors.

## Results

### Performance and Milk Composition

Milk composition was similar between treatments (Table 2). From period 1 to period 3, milk yield increased (1.03 vs. 1.34 kg/d), while milk fat (2.72 vs. 1.82 kg/d) and total solids (11.18 vs. 9.14 %) were reduced.

**Table 2:** Performance and milk composition from goats supplemented with rapeseed oil and sunflower oil (4% DM).

**Parameters**	**Diets**				**Treatment**	**Period**
	**Control**	**Rapeseed**	**Sunflower**	**SEM**	***P*-value**	***P*-value**
Body condition score	2.43	2.77	2.66	0.136	0.056	0.875
Body weight, kg	40.6	46.3	44.0	4.05	0.378	0.960
Milk yield, kg/d	1.03	1.26	0.92	0.133	0.056	0.049
Fat, %	2.017	2.270	2.260	0.174	0.356	0.014
Protein, %	2.607	2.417	2.823	0.163	0.155	0.744
Lactose, %	4.76	4.08	4.62	0.250	0.106	0.233
Casein, %	2.143	1.783	2.223	0.196	0.171	0.428
Total solids, %	10.19	9.35	10.56	0.453	0.122	0.025
Citric acid, %	0.123	0.100	0.096	0.124	0.180	0.070
Solids-not-fat, %	8.32	7.28	8.33	0.395	0.091	0.186
Urea, mg/100 mL	29.2	24.5	37.0	7.77	0.363	0.377
Free fatty acid, mEq/L	0.753	0.811	0.775	0.076	0.759	0.130
Freezing point depression, °C	566	500	565	42.0	0.306	0.367
Acidity, °Th	10.27	11.60	9.93	1.05	0.346	0.142
Density, kg/l	1027.27	1023.20	1027.57	1.56	0.085	0.265

*SEM, standard error of the mean*.

### Milk Fatty Acid Profile

Compared to the control and rapeseed oil, sunflower decreased C4:0 and both oils decreased C18:3n3 (Table 3). Rapeseed increased C18:2 cis9, trans11 compared to control and sunflower oil. From period 1 to period 3, contents of C12:0 (2.20 vs. 7.19 g/100g), C18:2n6 *trans* (0.35 vs. 1.04 g/100 g), C18:2 *cis*9, *trans*11 (0.139 vs. 0.198 g/100 g) were increased while C18:1 *trans*11 (1.16 vs. 0.49 g/100 g) and C18:1 *cis*9 (27.8 vs. 17.3 g/100 g), C18:3n3 (0.601 vs. 0.101 g/100 g), and MUFA (31.1 vs. 21.2 g/100 g) were decreased.

**Table 3:** Milk fatty acid profile from goats fed rapeseed oil and sunflower oil (g/100 g of fatty acid methyl esters).

		**Diet**				***P*-value**	
**Fatty acid (FA)**	**Control**	**Rapeseed**	**Sunflower**	**SEM**	**Treatment (T)**	**Period (P)**	**T × P**
C4:0	1.56^a^	1.67^a^	1.36^b^	0.08	0.025	0.138	0.084
C6:0	1.62	1.73	1.63	0.25	0.904	0.507	0.528
C8:0	2.19	2.12	2.16	0.34	0.978	0.153	0.549
C10:0	8.31	8.08	8.88	1.25	0.809	0.081	0.463
C11:0	2.72	1.62	2.59	0.98	0.515	0.613	0.921
C12:0	3.48	4.22	4.91	2.03	0.788	0.043	0.646
C14:0	6.85	7.91	6.47	1.51	0.639	0.028	0.843
C14:1	0.61	0.48	0.55	0.20	0.731	0.858	0.768
C15:0	1.07	0.77	0.19	0.30	0.454	0.010	0.124
C15:1	0.46	0.64	0.58	0.31	0.841	0.319	0.700
C16:0	27.3	24.1	29.8	2.94	0.233	0.108	0.298
C16:1	1.58	0.65	0.96	0.75	0.492	0.511	0.490
C17:0	0.70	0.52	0.52	0.24	0.709	0.991	0.728
C18:0	11.8	14.9	12.6	2.48	0.490	0.326	0.031
C18:1 *trans*11	0.56	0.89	0.44	0.29	0.345	0.014	0.691
C18:1 *cis*9	22.2	25.9	23.0	3.38	0.563	0.004	0.621
C18:2n6 *trans*	0.81	0.57	0.41	0.24	0.324	0.044	0.339
C18:2n6 *cis*	2.79	0.87	0.43	1.48	0.309	0.230	0.424
C20:1n9	0.66	0.24	0.16	0.30	0.277	0.087	0.241
C18:3n3	0.60^a^	0.20^b^	0.10^b^	0.15	0.041	<0.001	0.008
C18:2 *cis*9, *trans*11	0.13^b^	0.37^a^	0.19^b^	0.16	0.035	0.028	0.239
C18:3n6	0.09	0.22	0.15	0.10	0.480	0.058	0.671
C20:0	0.93	0.29	0.49	0.18	0.389	0.346	0.113
C22:1n9	0.06	0.09	0.08	0.09	0.930	0.888	0.167
C20:3n3	0.33	0.14	0.34	0.17	0.451	0.430	0.242
C20:5n3	0.6	0.32	0.08	0.37	0.339	0.314	0.364
C24:1n9	0.32	0.26	0.30	0.25	0.973	0.213	0.895
Σ Saturated FA	67.3	68.2	71.8	1.79	0.098	0.279	0.213
Σ Monounsaturated FA	26.4	29.2	26.2	2.43	0.442	0.002	0.557
Σ Polyunsaturated FA	5.32	2.33	1.53	1.95	0.206	0.090	0.190

*SEM, standard error of the mean; ^a, b^Means in the same row with different superscript letters are significantly different (P < 0.05)*.

### Milk Foodome

**Figure 1:**
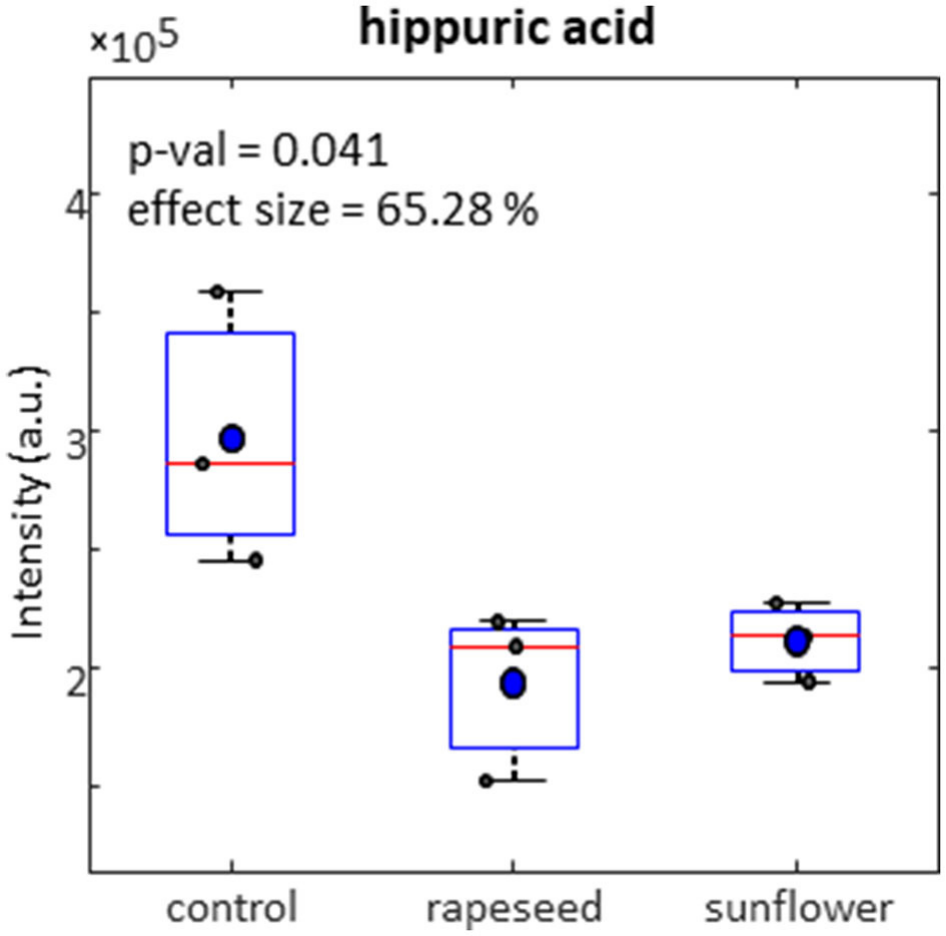
Relative concentration of hippuric acid in goat milk detected by ^1^HNMR spectroscopy across control, rapeseed oil and sunflower oil diets.

**Figure 2:**
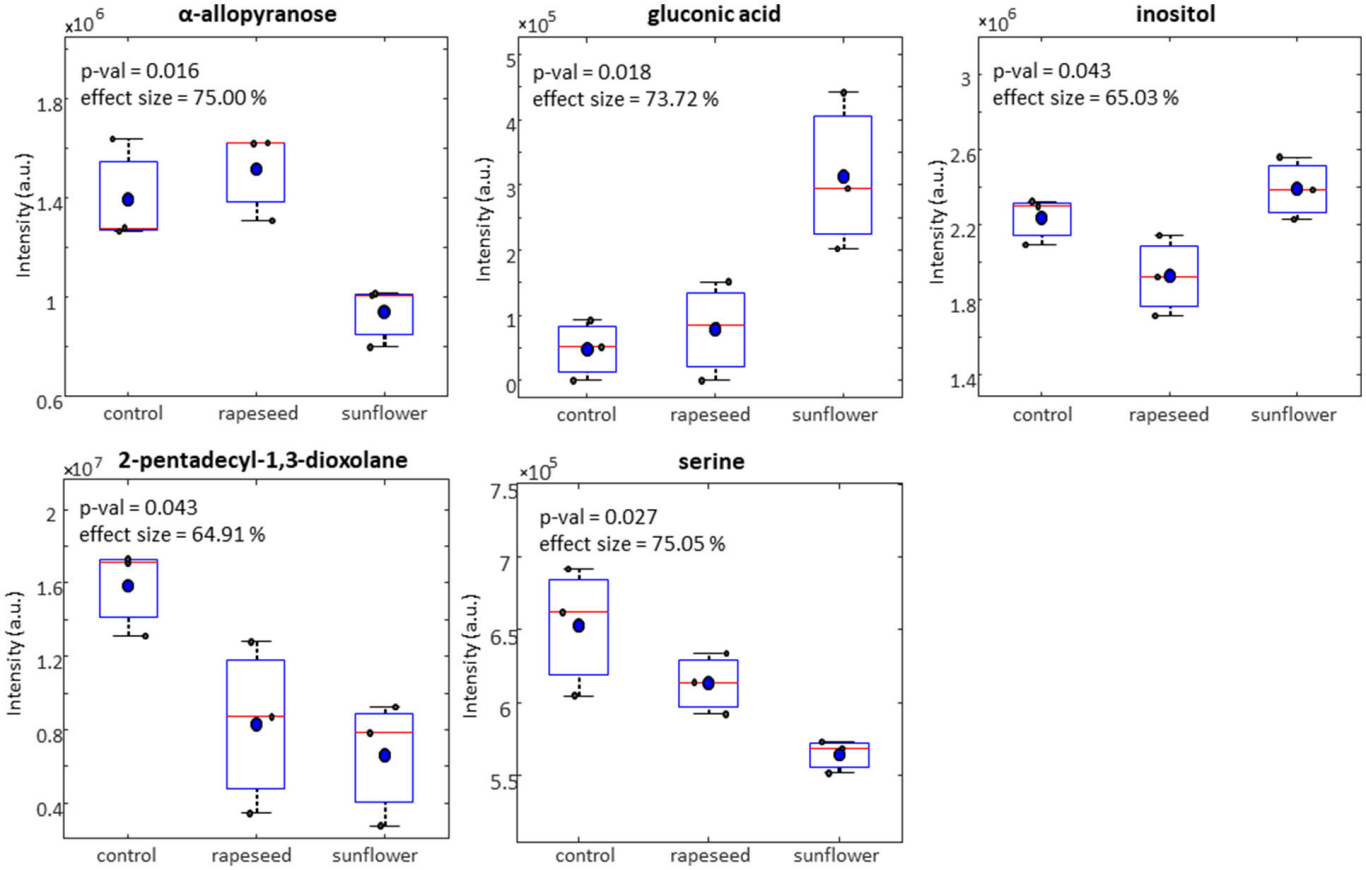
Relative concentrations of goat milk metabolites detected by GC-MS across control, rapeseed oil and sunflower oil diets.

Compared to control group, both rapeseed oil and sunflower oil groups had a lower levels of hippuric acid in milk samples measured by ^1^H NMR spectroscopy (Figure 1). GC-MS data revealed a total of five tentatively identified metabolites in milk, at level 2 based on the metabolomics standard initiative, including D-allopyranose, D-gluconic acid, inositol, 2-pentadecyl-1,3-dioxolane and serine. These metabolites were significantly different between the three treatments groups (Figure 2). The milk samples in the rapeseed oil group had a similar levels of α-D-allopyranose compared to the control group, while the samples in the sunflower oil group had a had significantly lower levels of this metabolite. Figure 2 shows a notable high level of D-gluconic acid and inositol in the milk samples from the sunflower oil group than the other two. The milk samples in the rapeseed oil group had a higher concentration of D-gluconic acid but lower inositol than the control group. 2-Pentadecyl-1,3-dioxolane and serine had a similar trend among the three groups, and the highest level of both metabolites was observed in the control group followed by the rapeseed oil group and the sunflower oil group.

### *In vitro* Fermentation Kinetics

Sunflower oil and rapeseed oil decreased total gas production at 24 and 48 h compared with control. Dry matter degradation was not affected by dietary treatments (Table 4). Individual volatile fatty acid proportions, total VFA, CO_2_, and CH_4_ were not affected by dietary treatments (Table 5).

**Table 4:** Total gas production at different time points and dry matter degradation (dDM).

	**ml/g DM**			**% DM**		
	**6h**	**9h**	**12h**	**24h**	**48h**	**dDM**
Control	20.71	34.97	51.40	103.48^b^	137.24^b^	63.45
Sunflower	22.15	33.79	48.76	93.69^a^	124.42^a^	63.34
Rapeseed	19.80	34.58	50.34	96.30^a^	125.83^a^	62.89
SEM	1.13	1.44	1.92	2.06	3.35	0.027
*P*-value	0.350	0.790	0.410	0.010	0.029	0.640

*SEM, standard error of the mean; ^a, b^Means in the same column with different superscript letters are significantly different (p < 0.05)*.

**Table 5:** Individual volatile fatty acid proportions (VFA; mol/100 mol), total VFA (mmol/L), CO_2_ mol and CH_4_ mol.

	**Acetic**	**Propionic**	**Isobutyric**	**Butyric**	**Valeric**	**Isovaleric**	**Caproic**	**Total VFA**	**CO_**2**_**	**CH_**4**_**
Control	22.63	6.54	0.67	3.65	0.90	0.81	0.21	35.41	18.42	11.50
Sunflower	23.36	6.77	0.68	3.72	0.91	0.82	0.21	36.47	18.95	11.85
Rapeseed	22.63	6.48	0.67	3.58	0.89	0.81	0.20	35.27	18.31	11.49
SEM	2.59	0.28	0.08	0.19	0.03	0.18	0.01	2.44	1.15	1.16
*P*-value	0.560	0.610	0.890	0.710	0.960	0.730	0.530	0.620	0.630	0.590

*SEM, standard error of the mean*.

## Discussion

It is important to mention that milk from small ruminants such as goats is of particular interest due to its unique FA configuration where caproic (C6:0), caprylic (C8:0), and capric (C10:0) represent from 15 to 18% of the goats milk fat (30). Therefore, research focused on improving goat milk fat composition is needed, especially in countries where not much research on small ruminants is done, as it is the case in Denmark.

The lack of effects on productive traits, such as milk yield, body conditions score, and body weight may be due to the relatively small amount of oil supplementation used in this study (4% DM). This agrees with previous studies where goats were fed with calcium soaps from palm, canola, and safflower oils (4) and when goats were supplemented with sunflower and linseed whole seeds (31). In those studies, oilseeds fed as calcium soaps or as whole seeds did not promote negative effects on overall animal productive traits and treatments were offered at <5% DM inclusion. This is the threshold level before dietary lipids start to cause digestive disruptions with concomitant effects on productivity (6). In this study, the choice of the amount of lipid included in the diet was conceived as a factor to promote a healthier milk FA profile without deleterious effects on productive parameters.

Milk fat was reduced over time while milk yield was increased which could have led to a dilution effect that was also observed for total solids. These results are positive as milk composition was not negatively affected by dietary oils. Normally, oils are added to goat diets to increase energy density but also to promote the formation of FA with potentially positive effects on human health (32). In this study, rapeseed oil increased the contents of C4:0 (butyric acid) and C18:2 *cis*9, *trans*11 (rumenic acid). Butyric acid is a short-chain FA that possesses antimicrobial activity and has been related to anti-diarrheic, antioxidant, anti-carcinogenic, and anti-inflammatory activities (33). Rumenic acid has been related to positive effects on serum lipid profile, blood glucose, insulin sensitivity, blood pressure, and cardiovascular disease risk factor in humans (34). Goat milk is mainly used for cheese manufacturing, and milk fat and protein are essential components needed for further processing (35). Therefore, our results show that using sunflower oil or rapeseed oil could be used in goat diets as a feeding strategy for modulating milk fat quality without negative effects on overall productive traits.

Dietary fat increases energetic efficiency in lactating ruminants, such as cows, and this is due to increased total energy intake. The energy intake is even greater than that coming from volatile fatty acids or protein. The increased energy can be directly incorporated into the products, as well as promoting nutrient partition toward milk production. However, large amounts of dietary fat in ruminant diets may possess deleterious effects on the rumen fermentation kinetics, decrease intestinal absorption, decrease contribution to total oxidation of nutrients, increase sensitivity to nutrient imbalance, causing reduced energy intake (36).

Production of rumen volatile FA has been used to estimate enteric methane production (37). In this study, we used our *in vitro* fermentation data to estimate methane production, however, this was not affected. It is important to note that the inhibitory response of fats to methane production depends on the concentration, type, the fatty acid composition of fats, and nutrient composition of diets (8). This study used 85% forage inclusion and 4% DM inclusion of dietary oils and probably the combination of both variables was not enough to produce changes during rumen fermentation. Further studies should use an *in vivo* approach to corroborate our findings on methane production.

It is well known that adding fats or oils to ruminant diets can affect the rumen microbiome and fermentation processes. Oil supplements can be toxic to the microbial community as is the case for species of gram-positive bacteria and ciliate protozoa or limit the microbial colonization of feed particles and the access of microbial enzymes to the substrates. Consequently, feed digestion (ruminal and total tract) may be adversely affected by the addition of lipids (38). At least at *in vitro* level, it has been suggested that the addition of vegetable oils to ruminant diets can improve feed efficiency and attenuate the environmental impact of ruminal fermentation contributing to more efficient, sustainable, and cleaner animal production (38).

Biohydrogenation of unsaturated fatty acids can compete with methanogenesis for metabolic H_2_ (39). It can be expected that considerable amounts of H_2_ will be used for the saturation of fatty acids provided with the oil supplemented diets, and thus diverted from methanogenesis (38). Our results on methane production should be corroborated by *in vivo* determinations of gas emissions or indirectly by studying the rumen microbiome with special regard to achaea populations.

Regarding milk foodome profile obtained from ^1^H NMR, the decrease of hippuric acid in milk obtained from rapeseed oil and sunflower oil compared to control could be correlated with pasture feeding (40), high dietary fiber and polyphenol-rich feeds (41), as well as negative energy balance status in cattle (42). Interestingly, hippuric acid has been proposed as a biomarker of pasture-derived milk (43) but in this study the presence of polyphenols in dietary oils (44) could be the reason for the increase of this metabolite. However, contents of polyphenols in dietary oils were not analyzed.

Glycine, glutamine, arginine, and serine appear to be the most abundant free amino acids in goat milk and milk formula, similar to the case for human milk or cow milk infant formula (45). In this study, with the use of ^1^H NMR spectroscopy, serine was found to be decreased by supplementing goats with either rapeseed or sunflower oils, however, no other significant effects were found in free amino acids that could represent a negative effect on the goat milk amino acid profile. However, further study is needed to validate our findings.

Gluconic acid was increased with sunflower oil. This organic acid is the product of glucose oxidation and has been used in the dairy industry to prevent or remove milk stone deposition and in the prevention of cloudiness in beverages (46). Further attention must be paid to the presence of this metabolite which was produced naturally when goats are fed with sunflower oil as it has a promising use for dairy processing. In this study, scyllo-inositol was higher in milk from sunflower oil. This metabolite is a stereoisomer of inositol and it has been reported as a promising therapeutic agent for Alzheimer's disease since it prevents the accumulation of beta-amyloid deposits, which are typical of this disease (47).

α-D-allopyranose was increased by rapeseed oil compared to sunflower (not compared to control) while 2-pentadecyl-1,3-dioxolane was decreased by sunflower oil compared to the control group. Information on the biological role of those metabolites related to goat milk is scarce. What is known is that lactose has partly protected derivates and those could yield precursors for the synthesis of higher oligosaccharides such as α-D-allopyranose (48). With regard to 2-pentadecyl-1,3-dioxolane, it has been related to long-chain cyclic acetals of glycerol (49) but at this point, it is difficult to understand its role in the milk metabolome of goats. It is important to note that when performing foodome profile using GC–MS and ^1^H NMR, milk samples were pooled by treatment and therefore, this data should be cautiously interpreted. Further studies should consider analyzing individual milk samples for foodome analysis.

## Conclusion

Overall, the use of sunflower oil or rapeseed oil at 4% DM inclusion did not compromise animal performance and milk composition.

## Data Availability Statement

The datasets presented in this study can be found in online repositories. The names of the repository/repositories and accession number(s) can be found in the article/Supplementary Material.

## Ethics Statement

The animal study was reviewed and approved by the present study was conducted in compliance with the Danish Ministry of Justice Law No. 474 (15 May 2014) concerning animal experimentation and the care of experimental animals. The Animal Ethics Institutional Review Board from the University of Copenhagen approved this study (ID 2021-08-PNH-008A).

## Author Contributions

EV-B-P and BK: conceptualization and methodology, validation, formal analysis, writing—original draft preparation, visualization, and supervision. EV-B-P, JK, YY, NP, RD, HHH, and BK: investigation and data curation. EV-B-P, JK, YY, NP, RD, HHH, LA, and BK: writing—reviewing and editing. All authors contributed to the article and approved the submitted version.

## Funding

This research was partly financed by the Cattle Group of the Section of Production, Nutrition and Health from the University of Copenhagen and Data plus project fund (Strategy 2013 funds) from the University of Copenhagen.

## Conflict of Interest

The authors declare that the research was conducted in the absence of any commercial or financial relationships that could be construed as a potential conflict of interest.

## Publisher's Note

All claims expressed in this article are solely those of the authors and do not necessarily represent those of their affiliated organizations, or those of the publisher, the editors and the reviewers. Any product that may be evaluated in this article, or claim that may be made by its manufacturer, is not guaranteed or endorsed by the publisher.
